# Cordycepin Ameliorates Synaptic Dysfunction and Dendrite Morphology Damage of Hippocampal CA1 *via* A1R in Cerebral Ischemia

**DOI:** 10.3389/fncel.2021.783478

**Published:** 2021-12-24

**Authors:** Zhao-Hui Chen, Yuan-Yuan Han, Ying-Jie Shang, Si-Yi Zhuang, Jun-Ni Huang, Bao-Yan Wu, Chu-Hua Li

**Affiliations:** ^1^School of Life Science, South China Normal University, Guangzhou, China; ^2^Panyu Central Hospital, Guangzhou, China; ^3^Ministry of Education (MOE) Key Laboratory of Laser Life Science, Institute of Laser Life Science, College of Biophotonics, South China Normal University, Guangzhou, China

**Keywords:** cordycepin, learning and memory (neurosciences), cerebral ischemia, synaptic function, dendritic morphology, adenosine A1 receptors

## Abstract

Cordycepin exerted significant neuroprotective effects and protected against cerebral ischemic damage. Learning and memory impairments after cerebral ischemia are common. Cordycepin has been proved to improve memory impairments induced by cerebral ischemia, but its underlying mechanism has not been revealed yet. The plasticity of synaptic structure and function is considered to be one of the neural mechanisms of learning and memory. Therefore, we investigated how cordycepin benefits dendritic morphology and synaptic transmission after cerebral ischemia and traced the related molecular mechanisms. The effects of cordycepin on the protection against ischemia were studied by using global cerebral ischemia (GCI) and oxygen-glucose deprivation (OGD) models. Behavioral long-term potentiation (LTP) and synaptic transmission were observed with electrophysiological recordings. The dendritic morphology and histological assessment were assessed by Golgi staining and hematoxylin-eosin (HE) staining, respectively. Adenosine A1 receptors (A1R) and adenosine A2A receptors (A2AR) were evaluated with western blotting. The results showed that cordycepin reduced the GCI-induced dendritic morphology scathing and behavioral LTP impairment in the hippocampal CA1 area, improved the learning and memory abilities, and up-regulated the level of A1R but not A2AR. In the *in vitro* experiments, cordycepin pre-perfusion could alleviate the hippocampal slices injury and synaptic transmission cripple induced by OGD, accompanied by increased adenosine content. In addition, the protective effect of cordycepin on OGD-induced synaptic transmission damage was eliminated by using an A1R antagonist instead of A2AR. These findings revealed that cordycepin alleviated synaptic dysfunction and dendritic injury in ischemic models by modulating A1R, which provides new insights into the pharmacological mechanisms of cordycepin for ameliorating cognitive impairment induced by cerebral ischemia.

## Introduction

Cerebral ischemia is a condition of significant mortality and morbidity that arises from a sudden loss of blood flow and a consequent failure to meet the demands of the brain for oxygen and glucose, which leads to functional and structural damages in different brain regions (Liang et al., 2015), such as the learning and memory impairments in the hippocampus (Gondard et al., 2019; Yao et al., 2019). The plasticity of the synaptic structure and function was associated with the cellular mechanism underlying learning and memory (Park et al., 2014; Bailey et al., 2015). Learning and memory impairments caused by cerebral ischemia were often closely related to the damages of synaptic transmission (Neumann et al., 2013) and dendritic morphological structure (Rojas et al., 2013; Zhu et al., 2017). Previous studies have reported that cognitive impairments after cerebral ischemia could be alleviated by improving synaptic plasticity (Li et al., 2017; Yu et al., 2018).

Cordycepin (3′-deoxyadenosine), a pharmacologically active ingredient derived from natural Chinese medicinal *Cordyceps militaris*, exhibits a variety of beneficial biological effects, including anti-tumor, antioxidant, anti-inflammatory, and antiviral activities (Jeong et al., 2011; Olatunji et al., 2016). In particular, the chemical structure of cordycepin is highly similar to that of adenosine (Yoon et al., 2018), which participates in the modulation of synaptic function through activating adenosine receptors (Chu et al., 2013). Moreover, the neuroprotective role of adenosine A1 receptors (A1R) in cerebral ischemia is widely accepted (Minelli et al., 2004; Bjorness et al., 2009). Meanwhile, it has been widely demonstrated that cordycepin exerted significantly neuroprotective effects (Yao et al., 2013; Peng et al., 2015) and protected against cerebral ischemic damage (Chen et al., 2017; Liu et al., 2017). Interestingly, our previous studies have revealed that cordycepin could ameliorate excitotoxicity-induced long-term potentiation (LTP) injury in rats by regulating the A1 receptor (Dong et al., 2019), and improving the conventional LTP and behavioral-LTP in healthy rats through modulating the A2A receptor (A2AR) (Cao et al., 2018; Han et al., 2019). Therefore, in the current study, we explore whether cordycepin ameliorates cognitive impairments induced by cerebral ischemia *via* reducing synaptic dysfunction and dendrite morphology damage and whether adenosine A1 receptors or A2A receptors are involved in the process.

## Materials and Methods

### Drugs and Chemicals

Cordycepin (>98% purity, C_10_H_13_N_5_O_3_) was provided by Prof. Hang-Hai Li, South China Normal University, China. Phentolamine was purchased from Shanghai Xudong Haipu Pharmaceutical company (China) and sodium nitroprusside was purchased from the Guangdong Hongyuan Group Pharmaceutical company (China). Other major chemicals used in the experiments included the antagonist of A1R, DPCPX (Tocris Company, Cat#0439), and the antagonist of A2AR, SCH58261 (Tocris Company, Cat# 2270), anti-Adenosine A1 receptor antibody (Abcam, Cat# ab82477), anti-Adenosine receptor A2A antibody (Abcam, Cat# ab3461), monoclonal Anti-γ-Tubulin antibody (Sigma-Aldrich, Cat# T6557), HRP-labeled Goat Anti-Rabbit IgG (H+L) (Beyotime biotechnology, Cat# A0208), and HRP-labeled Goat Anti-Mouse IgG (H+L) (Beyotime biotechnology, Cat# A0216).

### Animals and Treatments

Six to eight-week-old male Kunming mice (25–30 g) and 3-month-old male Sprague-Dawley (SD) rats (240–280 g) were obtained from the Sun Yat-sen University, China. The animals were housed at ~25°C and relative humidity of 50–55% in a light/dark cycle of 12/12 h, with free access to water and a rodent pallet diet. All animal procedures undertaken were approved by the Ethics Committee of Animal Research of the South China Normal University (No. SCNU-SLS2020-001) and were carried out in accordance with the principles outlined in the National Institute of Health (NIH) Guide for the Care and Use of Laboratory Animals (NIH Publications No. 8023, revised 1978). All animals were allowed to adapt to the circumstances for 3 days before the experiments. The investigators were blinded during experiments and data analysis.

In the *in vivo* experiments, the dose of 10 mg/kg cordycepin was referred to in the previous studies (Han et al., 2019). Previous studies have shown that the administration of cordycepin in advance has a protective effect on cerebral ischemia (Hwang et al., 2008; Cheng et al., 2011; Dong et al., 2019). And referring to the previous research in our laboratory (Cai et al., 2013; Cao et al., 2018; Han et al., 2019), 21-day cordycepin administration has the best effect on improving learning and memory. Therefore, pre-treatment was adopted and was adequate to study the role of cordycepin during ischemia. Efforts were made to reduce the number of animals used and to minimize their pain and discomfort by improving and perfecting the experimental procedures (e.g., intramuscular injection of 0.2 ml 40 U/ml penicillin after surgery to avoid infection).

### Measurement of Mean Arterial Blood Pressure

The rats were anesthetized by an intraperitoneal injection of sodium pentobarbital (40 mg/kg). The femoral artery and both common carotid arteries were separated and exposed carefully. A single lumen polyethylene tube (PE-50) which perfused 2 ml heparinized saline (100 ml 0.9% saline containing heparin) was catheterized in the V-shaped incision in the femoral artery and pushed forward about 2 cm. Cannulation was ligated distally. MABP was monitored with a PowerLab system ML4818 (AD Instruments Pty Ltd., Australia) throughout the whole experiment. After recording the baseline level for 30 min, the animals were injected with hypotensor (phentolamine and sodium nitroprusside) and their blood pressure was recorded for another 30 min.

### Establishment of Global Cerebral Ischemia

As described previously (Giuliani et al., 2009; Kocsis et al., 2014), the rats were anesthetized by an intraperitoneal injection of sodium pentobarbital (40 mg/kg i.p.) and fixed in a supine position. The body temperature of the rat was maintained at 37.0–37.7°C using a heater controller. After disinfecting with 75% alcohol, a midline incision was made in the neck of the rats. Both common carotid arteries (CCA) were separated and exposed. After the intraperitoneal injection of sodium nitroprusside or phentolamine, both CCA were occluded by artery clips for 15 or 20 min and reperfusion was achieved by removing the clips. The rats in the sham group were subjected to the same operation, except for bilateral CCA occlusion. After the operation was completed, the wound was sutured and an intramuscular injection of penicillin was administered.

### Electrophysiological Recording *in vivo*

The rats were anesthetized by an intraperitoneal injection of sodium pentobarbital (40 mg/kg) and fixed in a stereotaxic frame (RWD Life Science Cat# 68301). A single stimulating electrode (made with 0.1 mm nichrome wires, diameter 140 μm) was placed in CA3 at stereotaxic coordinates approximately AP 3.0–3.3 mm, L (R) 3.2–3.5 mm, H 3.8–4.2 mm. The evoked potential was extracellularly recorded with a monopolar electrode (Teflon-coated stainless-steel pins, 0.2 mm diameter, tip impedance, 2–5 M*Ω*) positioned in the pyramidal cell layer of ipsilateral CA1 at stereotaxic coordinates about AP 3.2–3.4 mm, L (R) 2.0–2.4 mm, H 2.8–3.0 mm. A small screw was inserted into the skull as the grounding electrode. After the appropriate stimulation and standard potential were measured, the electrode was fixed and sealed with medical dental powder. The rats were injected intramuscularly with penicillin to avoid infection after surgery.

Five days after surgery, the rats were placed in a quiet and insulated recording cage for about 15 min before the actual electrophysiological recording (conducted between 18:00 and 22:00). The intensity of the stimulus tested (a single pulse, 0.1–1.0 mA, 0.1 ms in duration, 1 min interval) was initially adjusted to produce 30–50% maximal population spikes (PS) on the first day of recording. Five average responses were used to estimate the PS amplitude (the distance between the negative minimum and the point that corresponds to the projection of the minimum on the line joining the two positive peaks) to minimize variations. The averaged PS was made for 3 consecutive days before the first day of Y-maze training and the testing stimulus intensity was kept at the same level in the following days. LTP is defined as an increase in PS amplitude by more than 30%.

### Y-Maze Behavioral Test

The y-maze is composed of three arms with the same size and 120° degree angle (45 cm long × 14 cm wide × 16 cm high), and the three arms are marked with “I,” “II,” and “III.” A 15-watt light is suspended at the end of each arm, and the whole device is placed in a dark environment. Light and dark discrimination experiments take advantage of the nature of rodents to avoid light and are usually used to test long-term working memory of rodents, and were referred to previous studies (Han et al., 2017, 2019). Firstly, the rat was put into the Y maze for 15 min of environmental adaptation. Then the rat was trained to switch to the randomly bright arm. Whenever the rat made a mistake (entering the dark arm), it was subjected to a brief electrical stimulation (30 V, 0.45 mA, begin after any arm lights up for 5 s) until it entered the bright arm. The rat was tested 20 times with an interval of 25 s every day during 6–7 days. Ninety percent correct ratio was defined as the learning standard. Rats that did not receive training but only recorded PS were regarded as the baseline group.

### Histological Assessment

After all the behavioral experiments were performed, the histological evaluation was assessed by hematoxylin-eosin (HE) staining. A portion of rats received sodium pentobarbital (40 mg/kg i.p.) and was transcardinally perfused with 0.1 M phosphate-buffered saline (PBS) until the liquid became clear, and then perfused with 4% paraformaldehyde. The brains were removed, post-fixed in the 4% paraformaldehyde at 4°C over 24 h. Afterward, the brains were dehydrated through a gradient sucrose solution and then embedded in paraffin. Horizontal sections (7 μm in thickness) at the levels of the middle of the hippocampus were cut by Microtome (Leica RM2255, Germany). The brain sections were deparaffined with xylene, rehydrated with gradient ethanol, and stained with hematoxylin and eosin in proper order. The histopathological changes of the hippocampus were visualized with an optical microscope (Leica, DM6) at 200× and 400× magnification. A rectangular area of equal size was randomly chosen from three different brain sections of one tissue sample for the average. The viable pyramidal cells in the CA1 region of the hippocampus were calculated according to the following criteria: the survived pyramidal cells are arranged densely and neatly, with rich and lightly stained cytoplasm, central nuclei, and clear nucleoli. Cells with concentrated red staining in the cytoplasm, shrinkage or karyopyknotic, dense, and deep staining of nuclear chromatin were considered dead cells.

### Golgi-Cox Staining

Golgi staining was performed according to the previous protocol (Han et al., 2019). The rats were anesthetized by intraperitoneal injection of sodium pentobarbital (50 mg/kg) and decapitated. The brains were rapidly removed and placed in the Golgi-Cox solution (5% potassium dichromate, 5% mercuric chloride, 5% potassium chromate, in distilled water) at 37°C in the dark for 7 days. Then, the brains were washed with distilled water and transferred into a protection solution (300 g/l sucrose in 0.01M PBS) for 3–5 days (changing the solution every day until the solution became clear). Brain samples were subjected to a full-automatic rotary microtome to obtain a dorsal hippocampal 150 μm coronal section and were pasted on a 3% gelatin-coated microscope slide. The sections were washed with distilled water, rinsed with 14% ammonia solution, and placed in the dark for 30 min. Next, the sections were washed again with distilled water and kept in 5% sodium thiosulfate in the dark. Finally, the sections were successively washed with distilled water, 70, 90, and 100% ethanol, and then immersed in xylene. The dendrites and spines in the CA1 regions were imaged using a light microscope (Leica DM6 FS, Germany) at both low (20×) and high (40×) magnifications, respectively. Neurons should be selected according to the previous description (Zaqout and Kaindl, 2016; Zhong et al., 2019). Sholl analysis plugin of Image J software was employed to assess the length and complexity of the dendrites. The dendritic spine density is expressed as the number of spines/dendritic segment lengths per 10 μm.

### Western Blotting

The hippocampus tissue was separated and homogenized in lysis buffer (0.32 M sucrose, 1 mM EDTA, 10 mM HEPES, 1 mg/ml BSA, 0.1 mM PMSF, pH 7.4). After the homogenate was centrifuged at 3,000 g at 4°C for 10 min, the supernatant was centrifuged at 16,000 g at 4°C for 60 min, and the precipitate was re-suspended in 5% (W/V) SDS. Next, the protein extracts were electrophoretically separated by 10% SDS polyacrylamide gel electrophoresis, and then the gel was transferred to a 0.45 μm nitrocellulose (NC) membrane at 4°C. After blocking non-specific sites with TBST (Tris Buffered Saline with Tween 20) containing 5% defatted dried milk, the membranes were incubated with the antibodies of A1R (1:1,000) and A2AR (1:1,000) overnight at 4°C, and γ-tubulin antibody (1:5,000) blotting was used as a loading control. The membranes were incubated with goat anti-mouse and goat anti-rabbit secondary antibodies in 5% defatted dried milk with TBST at room temperature for 1 h. Analysis was performed using an ECL chemiluminescence detection system (Millipore, USA) and the band intensity was quantified with Image J software (National Institutes of Health, Bethesda, MD, USA).

### Hippocampal Slices Preparation and Electrophysiological Recording *in vitro*

The method of hippocampal slices preparation was performed as described previously (Jiang et al., 2015). Briefly, the brains of the mice were removed quickly and placed in the ice-cold artificial cerebrospinal fluid (ACSF) with the following composition (mM): 117 NaCl, 4.7 KCl, 1.2 NaH_2_PO_4_·2H_2_O, 25 NaHCO_3_, 11 D-Glucose, 5 MgSO_4_·7H_2_O, 2.5 CaCl_2_ bubbled with 95% O_2_/5% CO_2_ (pH 7.35–7.45). Acute hippocampal slices (350–400 μm thickness) were prepared and maintained in an interface recording chamber containing preheated ACSF at 31 ± 0.5°C. Recordings began after 90 min of incubation.

**Figure 1:**
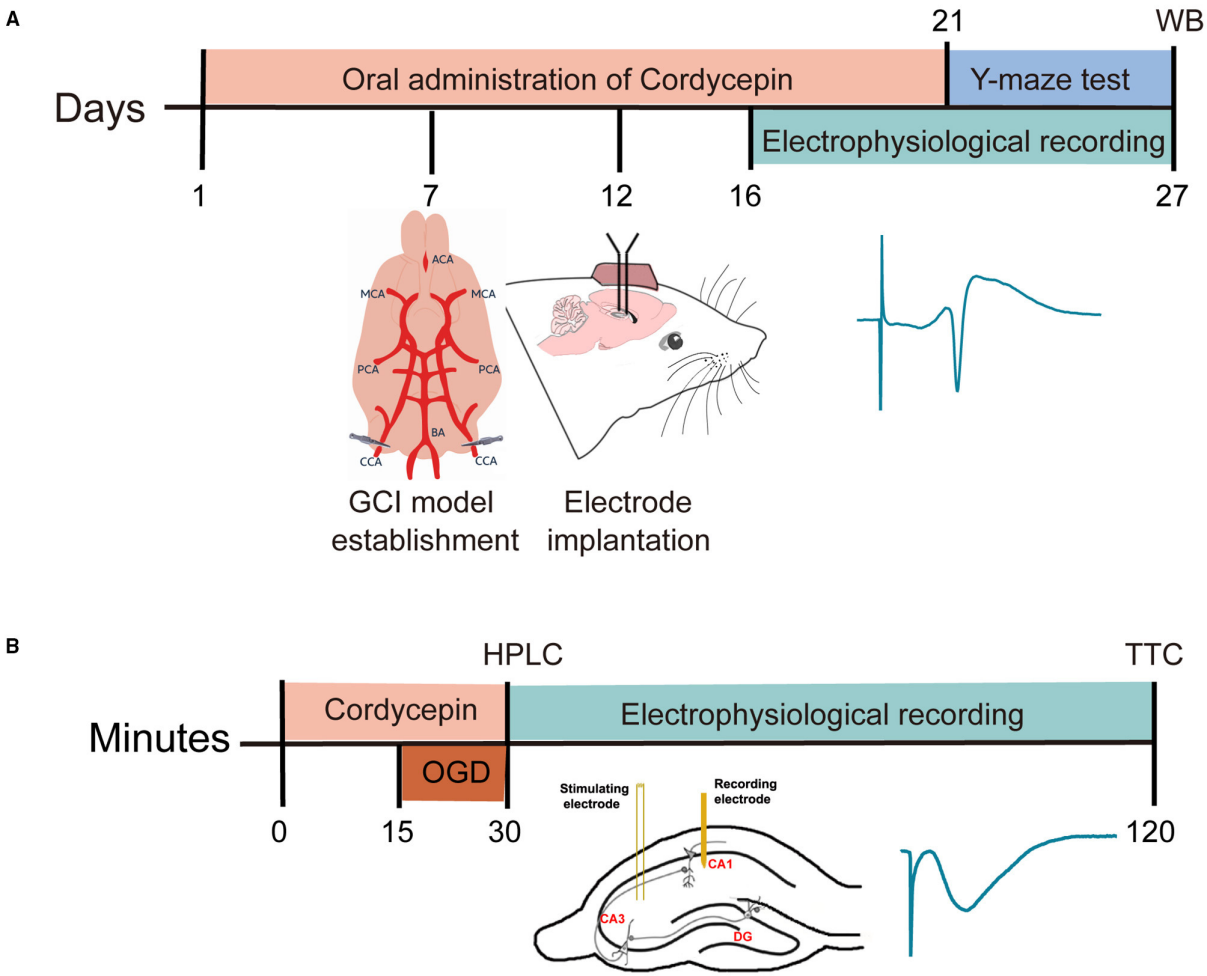
Time-line diagram of research design. **(A)** The timeline diagram of the *in vivo* experiments. **(B)** The timeline diagram of the *in vitro* experiments.

The field excitatory postsynaptic potentials (fEPSP) were recorded from CA1 stratum pyramidal cells using the recording electrode, a single glass pipette filled with 2M NaCl (resistance of 2–5 MΩ). A bipolar stimulation electrode (twisted nichrome wire, ϕ 140 μm) was positioned at schaffer collateral-commissural projection, in the CA3 region. Once the slope of fEPSP has been maximized and stabilized, an input/output (I/O) curve was induced. The optimal stimulation intensity (0.3–1.0 mA, 0.1 ms induration) was set at about 30% of the maximal I/O value through the experiment. After 15–20 min of recording the baseline stabilization, the control was switched from ACSF to oxygen-glucose deprivation (OGD) or cordycepin-containing ACSF. The OGD group was perfused with glucose-free ACSF gassed with 95% N_2_/5% CO_2_ for 15 min, and then the hippocampus slices were moved to the normal ACSF for 90 min. A total of 20 μg/ml cordycepin was perfused for 15 min in advance and OGD was applied with the perfusion of cordycepin. The timeline diagram of the research design is shown in Figure 1B. Three average responses were taken to estimate the fEPSP slope. Data were obtained and analyzed by LTP230D software.

### High-Performance Liquid Chromatography and 2, 3, 5-Triphenyl-Tetrazolium Chloride Staining

At the end of OGD or cordycepin-treatment, the perfusion fluid was collected for 10 min, then lyophilized, reconstituted with 400 μl ddH_2_O, centrifuged at 4°C (12,000 rpm, 10 min), and the supernatant was detected with a poroshell 120 EC-C18 column (4.6 × 50 mm, 2.7 μm) at a rate of 0.6 ml/min by a high-performance liquid chromatography (HPLC) instrument (Agilent Technologies 1,260 Infinity, USA). Adenosine was detected at 260 nm, 10 μl injection volume, and the mobile phases were composed of water and acetonitrile.

At the end of incubation, the hippocampal tissues were stained with 2% TTC in the dark for 1 h and then washed with saline. Afterward, hippocampal tissues were extracted with extraction solvent (ethanol and DMSO at 1:1, 1:20 w/v) for 24 h in the dark, the absorbance at 490 nm (OD_490_) was measured using a spectrophotometer (UV2550, Shimadzu, Japan). Tissue injury ratio was expressed as follows: Tissue injury ratio (%) = (1 – OD_490_ nm injury/OD_490_ nm control) × 100%. TTC staining is a reaction between TTC and succinate dehydrogenase in the mitochondria of living cells to produce red Formazan. While cells in the ischemic-infracted tissue lost the enzyme and do not have this reaction, thus remain pale. Dimethyl sulfoxide (DMSO) can dissolve red formazan generated in cells, and its light absorption was measured by spectrophotometer at 490 nm wavelength. According to the measured absorbance (OD value), the viability of cells in tissues can be determined, the larger the OD value, the smaller the tissue damage rate.

### Statistical Analysis

GraphPad Prism 8 and SPSS 14.0 software packages (Chicago, IL, USA) were used for graphing and statistical analysis. The data were expressed as mean ± SEM. Data conforming to a normal distribution (*p* > 0.05 in K-S test) and homogeneity of variance (*p* > 0.05 in Levene's test) were analyzed by one-way ANOVA, followed by Dunnett's *post-hoc* analysis for multiple comparisons, and the student's test was used to compare the two groups. Data that did not meet the above conditions were analyzed using the Kruskal-Wails test. For all comparisons, *p* < 0.05 was the standard for statistically significant differences. The ratio of the criteria was expressed as a percentage and evaluated by the chi-square test.

## Results

The timeline diagram of the research design was shown in Figure 1. The present study included *in vivo* and *in vitro* experiments. Cordycepin was administered 10 mg/kg intragastric twice a day (morning and afternoon) for 21 days. In the *in vitro* experiments, we focused on the protective effect of cordycepin on OGD by using mice brain slices.

### GCI Model Was Suitable for Evaluating Learning and Memory Impairments

**Figure 2:**
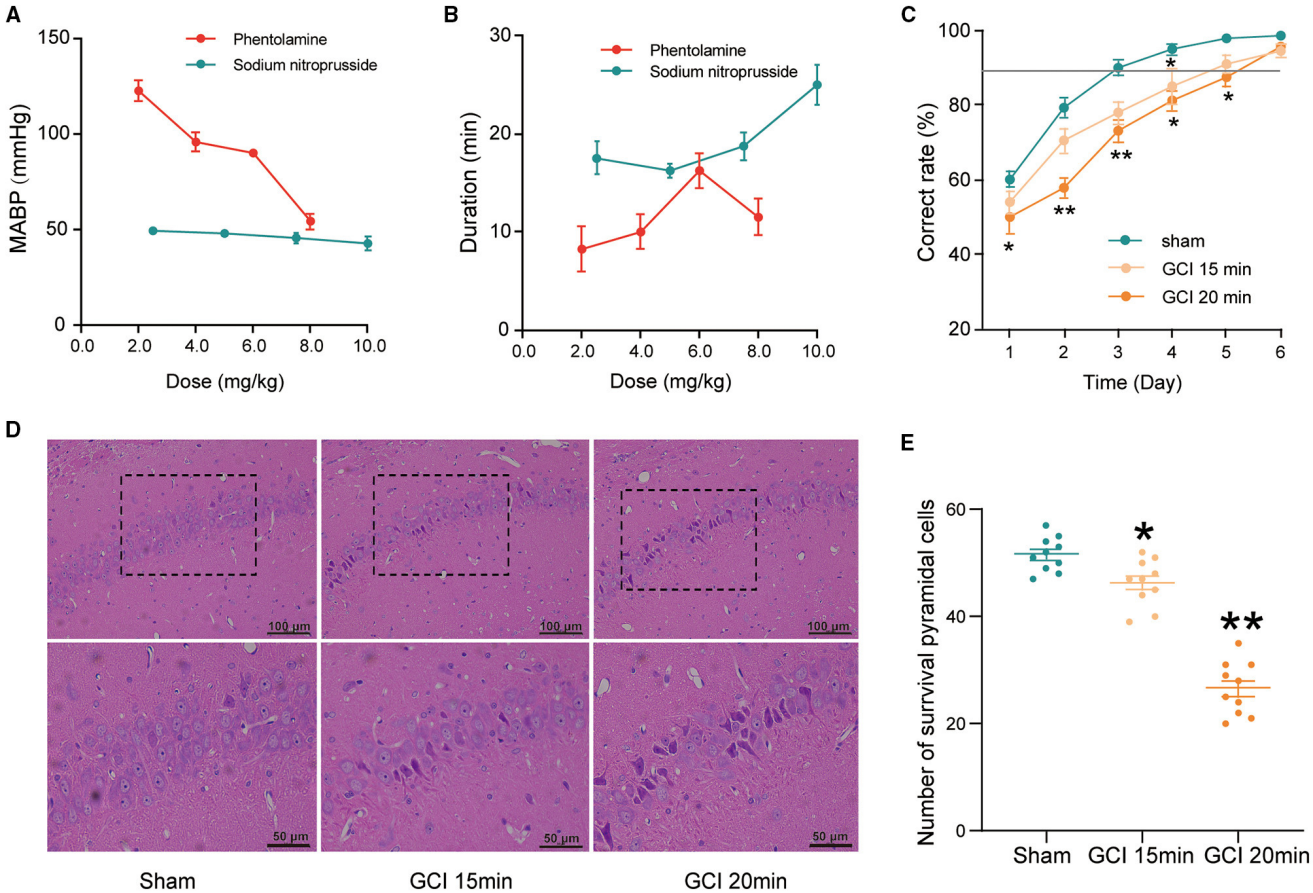
The establishment of the global cerebral ischemia (GCI) rat model. **(A)** Mean arterial blood pressure (MABP) in rats with different doses of phentolamine and sodium nitroprusside. Each group *n* = 5. **(B)** The duration of hypotensive effects with different doses of phentolamine and sodium nitroprusside. Each group *n* = 5. **(C)** Effects of ischemia time on the correct rate in Y-maze performance of rats. Each group *n* = 10. **(D)** Representative hematoxylin-eosin (HE)-stained sections of hippocampal CA1 regions (200×/400×, scale bar: 100/50 μm). The survived pyramidal cells are arranged densely and neatly, with rich and lightly stained cytoplasm, central nuclei, and clear nucleoli. Cells with concentrated red staining in the cytoplasm, shrinkage or karyopyknotic, and dense and deep staining of nuclear chromatin were considered dead cells. **(E)** The number of survived neurons counted in the CA1 regions. Each group *n* = 10/5 (brain sections/rats). Data were expressed as mean ± SEM; One-way ANOVA, *p < 0.05, **p < 0.01.

To establish a GCI model suitable for evaluating learning and memory impairments, two antihypertensive drugs and two ischemic times for two-vessel occlusions were assessed. As shown in Figures 2A,B, the four dosages (2, 4, 6, and 8 mg/kg) of phentolamine all could not reduce the MABP of rats below 50 mm Hg. According to Smith, MABP must be maintained at 50 mm Hg during ischemia (Smith et al., 1984). However, the MABP in the 2.5 and 5 mg/kg sodium nitroprusside groups were 49.4 ± 1.4 and 48.0 ± 2.0 mm Hg, respectively, and lasted for ~18 min. Moreover, 7.5 and 10.0 mg/kg dosages of sodium nitroprusside also could decrease MABP to 45.6 ± 2.6 and 42.7 ± 3.3 mm Hg and lasted for about 20 min but increased the risk of death (data not shown). Therefore, 2.5 mg/kg sodium nitroprusside was selected because of appropriate hypotension and maintenance time.

Neuronal damage in the hippocampus was proportional to the ischemic duration (Yu et al., 2012). As shown in Figure 2D, the neurons in the hippocampal CA1 region, in the ischemia 15 and 20 min groups, were extensively damaged and evidenced by neuronal shrinkage, karyopyknosis of nuclei, and irregular arrangement. Furthermore, the number of survived pyramidal cells was significantly reduced in the ischemia 15 min and ischemia 20 min group compared with the sham group (*F*_2, 27_ = 96.23, *P* < 0.05; Figure 2E).

Light-dark discrimination training in Y-maze was used to assess learning and memory impairment. As shown in Figure 2C, the correct ratio in the ischemia 20 min group was significantly lower than that of the sham group at days from 1 to 5 (day 1: *F*_2,27_ = 2.762, *P* = 0.049; day 2: *F*_2,27_ = 10.218, *P* < 0.0001; day 3: *F*_2,27_ = 6.577, *P* = 0.003; day 4: *F*_2,27_ = 3.067, *P* = 0.039; day 5: *F*_2,27_ = 2.919, *P* = 0.042; Figure 2C). Collectively, 2.5 mg/kg sodium nitroprusside combined with two vessel occlusions for 20 min may obtain an appropriate model to evaluate cognitive impairments caused by cerebral ischemia.

### Cordycepin Ameliorated Behavioral LTP Suppression and Learning and Memory Impairments in GCI Rats

**Figure 3:**
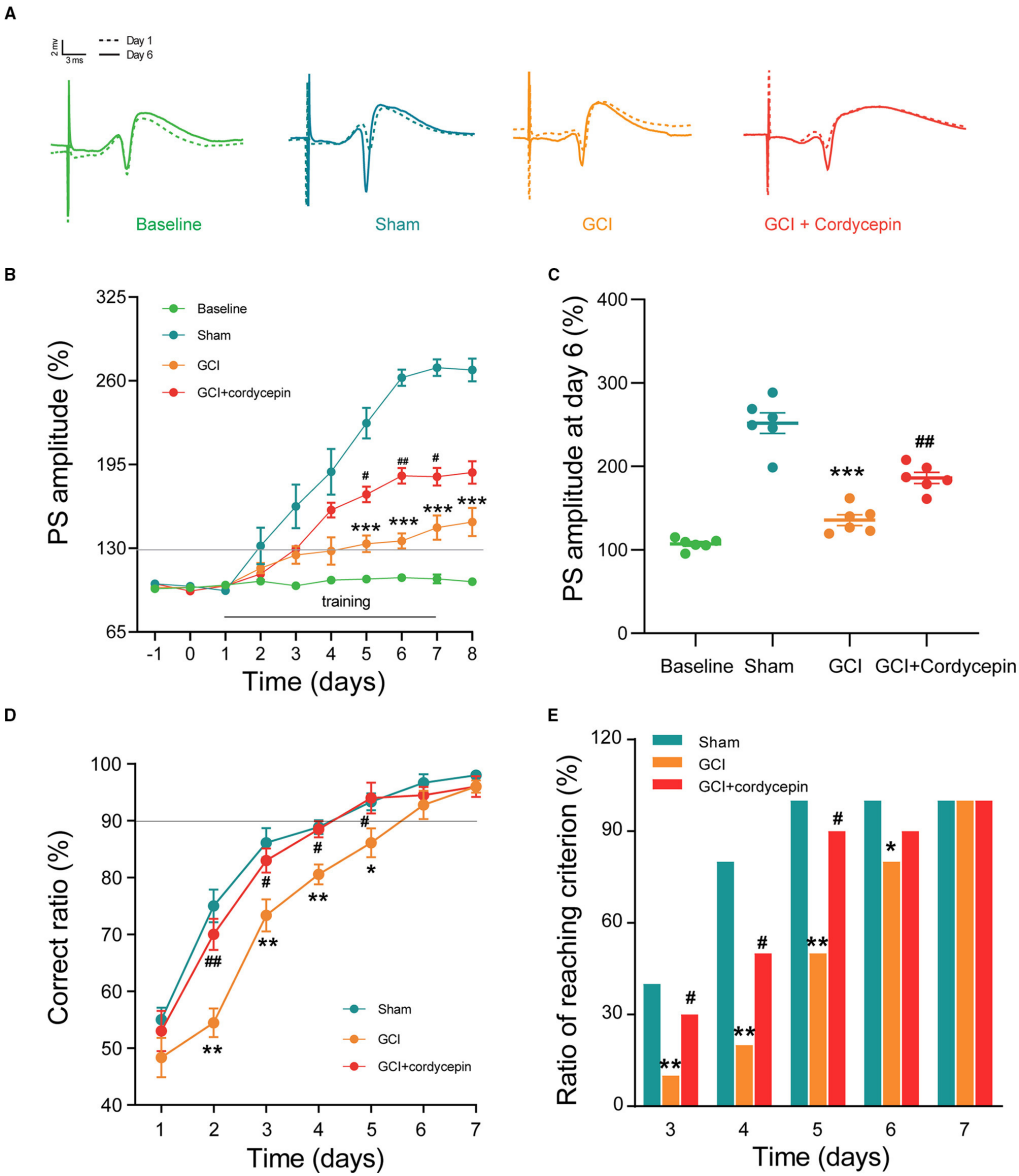
Cordycepin improved the magnitude of behavioral-long-term potentiation (LTP) and learning and memory in GCI rats. **(A)** The original population spikes (PS) at day 1 and 7; Dotted track: PS original potential at day 1; Solid track: PS original potential at day 7. **(B)** Changes in PS amplitude during 10 days training of Y maze. Each group *n* = 6 rats. **(C)** Comparison of PS amplitude at day 6 of training. Each group *n* = 6 rats. **(D)** The correct ratio in the Y maze test for 7 consecutive days. Each group *n* = 10 rats. **(E)** The ratio of meeting learning standards from day 3 to day 7, the learning standard was defined as the accuracy of 90% or above. Each group *n* = 10 rats; One-way ANOVA and chi-square test, *p < 0.05, **p < 0.01, ***p < 0.001, Sham vs. GCI; ^**#**^*p* < 0.05, ^**##**^*p* < 0.01, GCI vs. GCI + cordycepin.

In the *in vivo* electrophysiological experiments, PS was recorded to evaluate synaptic plasticity in GCI rats (Figure 3A). As shown in Figure 3B, the PS amplitude remained stable in the baseline group without Y-maze training. Consecutively, behavioral training caused a gradual potentiation in the PS amplitude. Compared with the sham group, the potentiation of PS amplitude after training was significantly reduced in the GCI group at day 5–8 (day 5: *F*_3,20_ = 3.293, *P* < 0.0001; day 6: *F*_3,20_ = 1.629, *P* < 0.0001; day 7: *F*_3,20_ = 1.083, *P* < 0.0001; day 8: *F*_3,20_ = 1.298, *P* < 0.0001; Figures 3B,C). However, 10 mg/kg cordycepin restored PS amplitude potentiation at day 5–7 as compared with the GCI group (day 5: *F*_3,20_ = 3.293, *P* = 0.021; day 6: *F*_3,20_ = 1.629, *P* = 0.001; day 7: *F*_3,20_ = 1.083, *P* = 0.0163; Figures 3B,C), suggesting that cordycepin could ameliorate the suppression of behavioral-LTP in GCI rats. cordycepin significantly.

As shown in Figure 3D, in the three groups, the correct ratio was raised gradually during 7 days of training and then reached the learning criterion of 90% correct ratio. However, the correct ratio at day 2–5 (day 2: *F*_2,27_ = 4.621, *P* = 0.01; day 3: *F*_2,27_ = 5.028, *P* = 0.011; day 4: *F*_2,27_ = 9.314, *P* = 0.001; day 5: *F*_2,27_ = 4.040, *P* = 0.022; Figure 3D) and the ratio of reaching criterion at day 3–6 (chi-square-test; day 3: *P* = 0.001; day 4: *P* = 0.006; day 5: *P* = 0.007; day 6: *P* = 0.001; Figure 3E) were significantly lower in the GCI group as compared with those in the sham group, which indicated that cognitive deficits existed in the GCI group. Similarly, cordycepin significantly enhanced the correct ratio at day 2–5 (day 2: *F*_2,27_ = 4.621, *P* = 0.048; day 3: *F*_2,27_ = 5.028, *P* = 0.049; day 4: *F*_2,27_ = 9.314, *P* = 0.005; day 5: *F*_2,27_ = 4.040, *P* = 0.046; Figure 3D) and the ratio of reaching criterion at training day 3–5 (chi-square test; day 3: *P* = 0.05; day 4: *P* = 0.011; day 5: *P* = 0.022; Figure 3E), suggesting that cordycepin could partially improve the learning and memory impairments in GCI rats.

### Cordycepin Alleviated the Damage of Dendritic Morphology in the Hippocampal CA1 Area of GCI Rats

**Figure 4:**
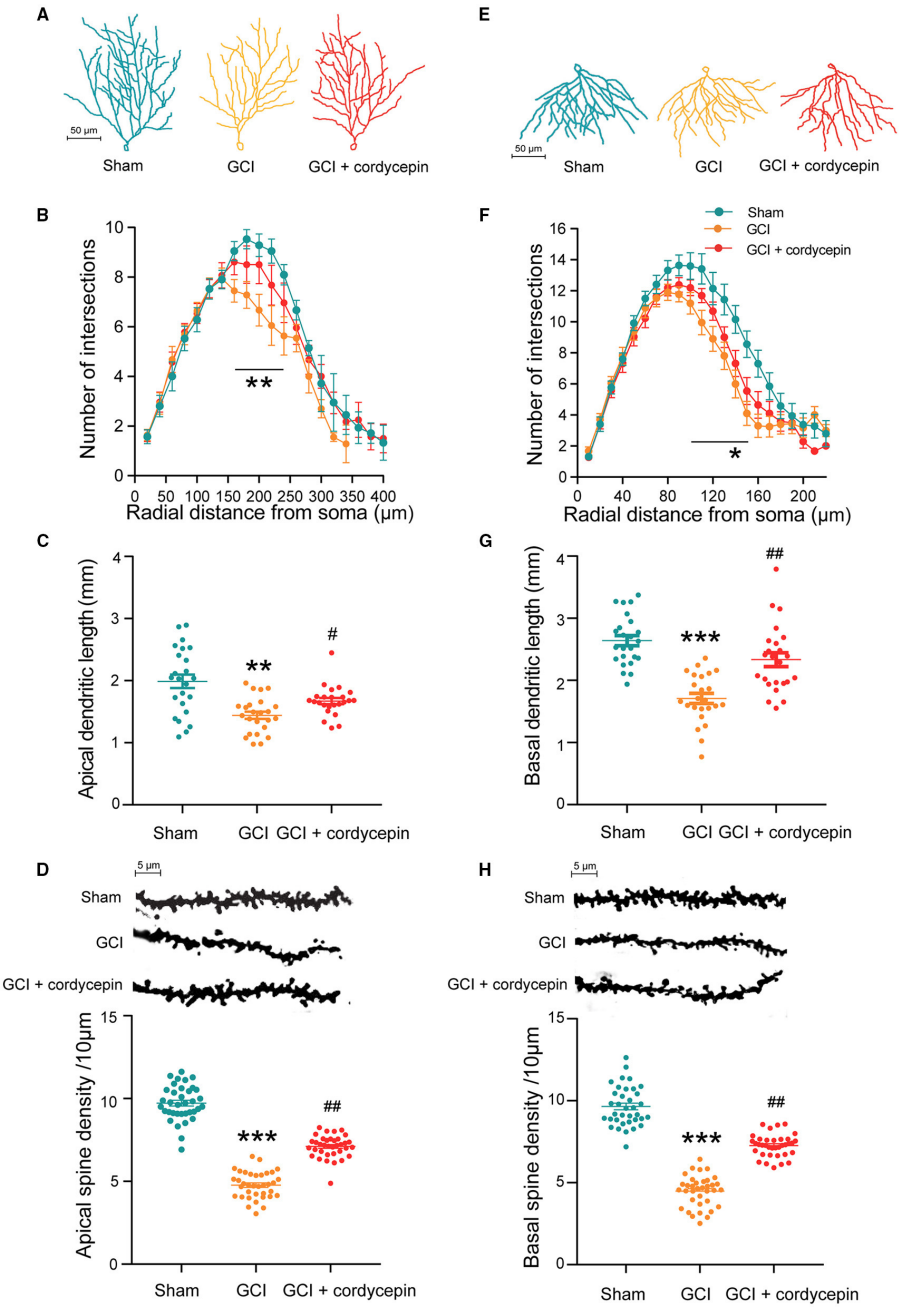
Cordycepin alleviated the damage of dendritic morphology in the hippocampal CA1 area of GCI rats. **(A,E)** Representative trajectories of the apical and basal dendrites of pyramidal neurons in the CA1 region of the hippocampus. **(B,F)** Intersection points of pyramidal neurons at the apical and basal dendritic branches in CA1 region. Apical: Sham group *n* = 20 neurons/6 rats, GCI group *n* = 22 neurons/6 rats, GCI + cordycepin *n* = 28 neurons/6 rats. Basal: Sham group *n* = 23 neurons/6 rats, GCI group *n* = 21 neurons/6 rats, GCI + cordycepin *n* = 26 neurons/6 rats. **(C,G)** Comparison of the total length between the apical and basal dendrites of pyramidal neurons in the CA1 region. Each group *n* = 24 neurons/6 rats. **(D,H)** Representative images and density of apical and basal dendritic spines of pyramidal neurons in CA1 region. Each group *n* = 36 dendrites/6 rats. Data were expressed as mean ± SEM; One-way ANOVA, N.S., not significant, *p < 0.05, **p < 0.01, ***p < 0.001, Sham vs. GCI; ^**#**^*p* < 0.05, ^**##**^*p* < 0.01, GCI vs. GCI + cordycepin.

To study the effect of cordycepin on the dendritic morphology of GCI rats, we analyzed the dendritic characteristics of the pyramidal neurons in the hippocampal CA1 area (total dendritic length, dendritic branches and dendritic spine density). As shown in Figures 4A,D,E,H, the apical dendrites, basal dendrites and dendritic spines from pyramidal neurons were clearly shown. Compared with the sham group, the number of intersections of apical dendrites at distances of 160–240 μm (160 μm: *F*_2,67_ = 1.327, *P* = 0.0167; 170 μm: *F*_2,67_ = 4.038, *P* = 0.0376; 180 μm: *F*_2,67_ = 4.639, *P* = 0.0107; 190 μm: *F*_2,67_ = 2.308, *P* = 0.0903; 200 μm: *F*_2,67_ = 2.109, *P* = 0.0055; 210 μm: *F*_2,67_ = 1.292, *P* = 0.8842; 220 μm: *F*_2,67_ = 2.338, *P* = 0.0037; 230 μm: *F*_2,67_ = 1.386, *P* = 0.0038; 240 μm: *F*_2,67_ = 3.764, *P* = 0.017; Figure 4B) and basal dendrites at distances of 100–150 μm (100 μm: *F*_2,67_ = 2.984, *P* = 0.0276; 110 μm: *F*_2,67_ = 3.489, *P* = 0.0033; 120 μm: *F*_2,67_ = 1.386, *P* = 0.0121; 130 μm: *F*_2,67_ = 0.8153, *P* = 0.0072; 140 μm: *F*_2,67_ = 0.06036, *P* = 0.0051; 150 μm: *F*_2,67_ = 0.4229, *P* = 0.0012; Figure 4F), the total length of apical (*F*_2,69_ = 26.32, *P* < 0.0001; Figure 4C) and basal dendrites (*F*_2,69_ = 12.66, *P* < 0.01; Figure 4G), and the density of dendritic spines in apical (*F*_2,105_ = 299.5, *P* < 0.0001; Figure 4D) and basal dendrites (*F*_2,105_ = 256.3, *P* < 0.0001; Figure 4H) were significantly reduced in the GCI group. Compared with the GCI group, cordycepin treatment could significantly reverse the decrease in the total length of dendrites (apical: *F*_2,69_ = 26.32, *P* < 0.0001, Figure 4C; basal: *F*_2,69_ = 12.66, *P* = 0.04, Figure 4G) and the spine density in both apical and basal dendrites (apical: *F*_2,105_ = 299.5, *P* < 0.0001, Figure 4D; basal: *F*_2,105_ = 256.3, *P* < 0.0001, Figure 4H) of GCI rats, but did not change the number of dendritic intersections. These results suggested that cordycepin could alleviate the damage of dendritic morphology in the hippocampal CA1 area of GCI rats.

### Cordycepin Reversed the Reduction of A1R in GCI Rats

**Figure 5:**
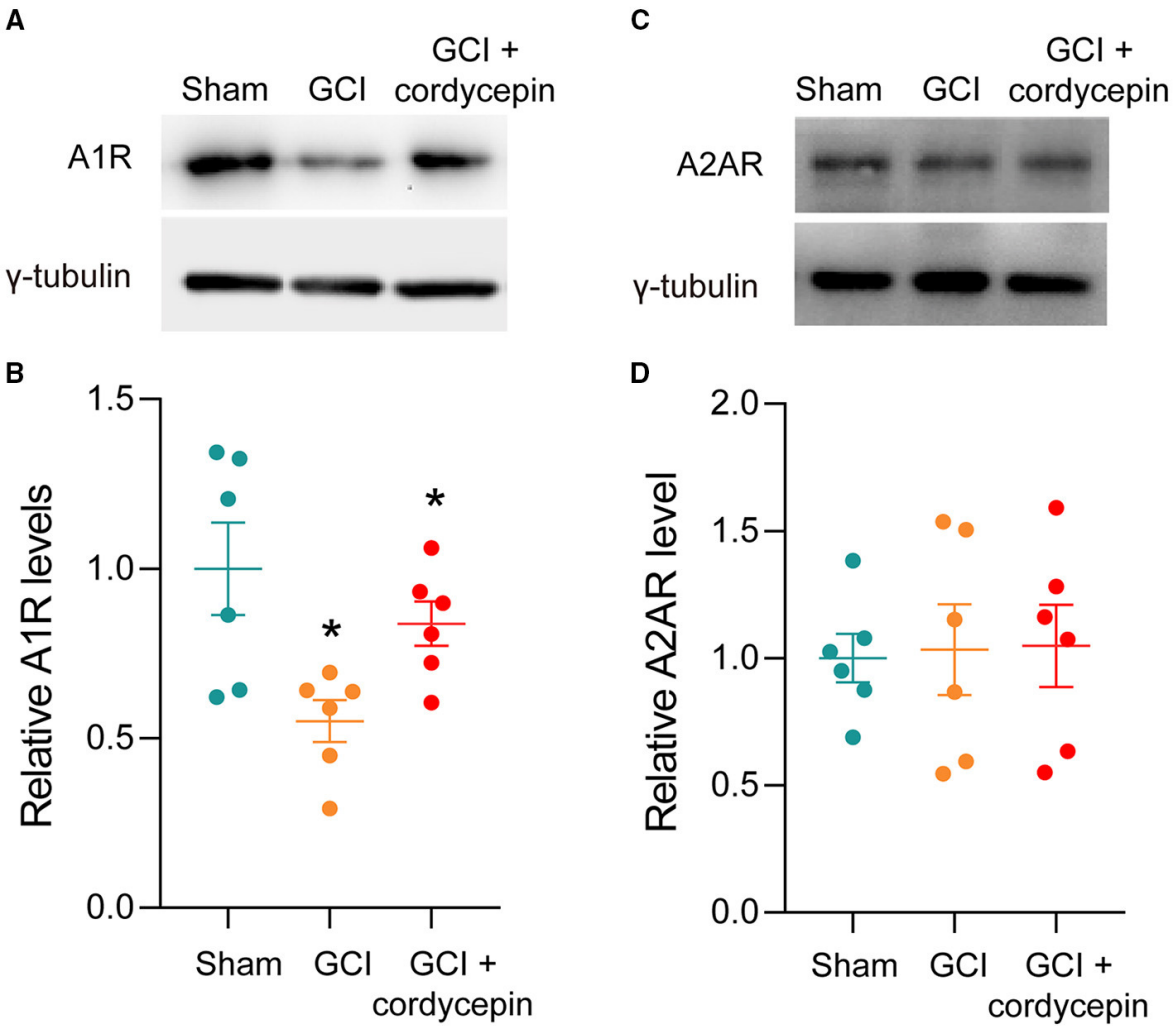
Cordycepin abolished the decline in the level of A1R in GCI rats. **(A,C)** Representative blots of adenosine A1 and A2A receptors level in hippocampus of rats. **(B,D)** Quantitative analysis of adenosine A1 and adenosine A2A receptors level in hippocampus of rats. Each group *n* = 3 rats. Data were expressed as mean ± SEM; One-way ANOVA, N.S., not significant, *p < 0.05.

The representative bands of A1R and A2R were shown in Figures 5A,C. Compared to the sham group, the level of A1R was significantly reduced in the GCI group (*P* = 0.0134), and 10 mg/kg cordycepin treatment increased the level of A1R in the hippocampus (*P* = 0.0399; Figure 5B). However, there were no differences in the A2AR level among the three groups (*F*_2,15_ = 0.0278, *P* = 0.9725; Figure 5D). These results demonstrated that cordycepin could modulate the level of A1R but not A2AR in GCI rats.

### Cordycepin Protected Against Hippocampal Slices Injury Induced by OGD and Increased the Content of Adenosine After OGD

**Figure 6:**
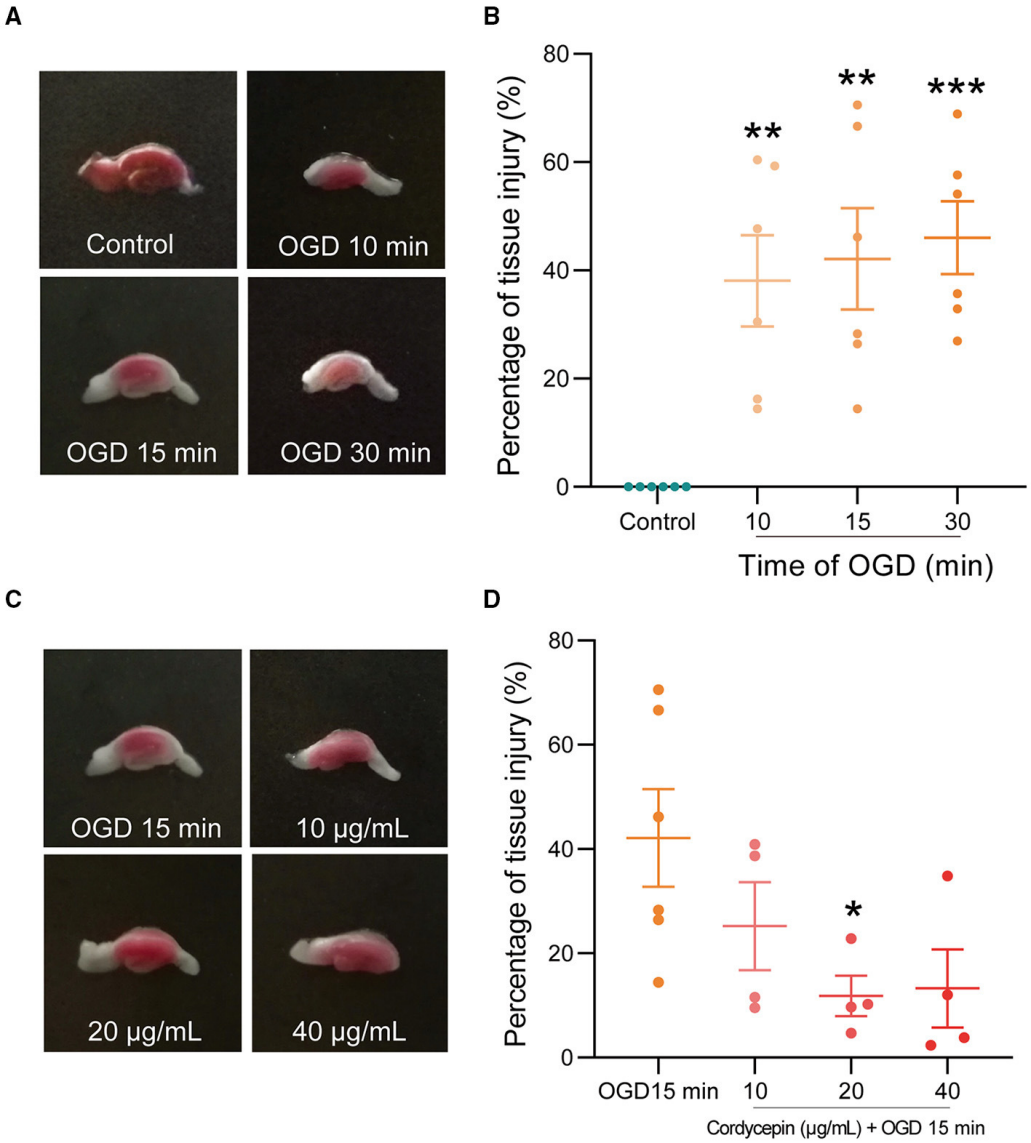
Cordycepin reduced hippocampal slices injury caused by OGD. **(A,B)** Representative triphenyl-tetrazolium chloride (TTC) staining images on hippocampal tissue after OGD for 10, 15, and 30 min, respectively. Each group *n* = 6 mice. **(C,D)** Cordycepin protected hippocampal tissue against OGD-caused injury. Tissue injury ratio (%) = (1 – OD_490_ nm injury/OD_490_ nm control) × 100%. OGD group *n* = 6 mice; Cordycepin group *n* = 4 mice. Data were expressed as mean ± SEM; one-way ANOVA, *p < 0.05, **p < 0.01, ***p < 0.001.

Oxygen-glucose deprivation (OGD) was chosen as an appropriate model for studying the molecular mechanisms of ischemic conditions, offering the advantages of the integrity of local neuronal circuits but the exclusion of complex parameters *in vivo* (Ziemka-Nalecz et al., 2013). As shown in Figures 6A,B, the OGD groups showed a pale, infarcted region compared with the control group, whose hippocampal slices were stained completely red by TTC. OD_490_ calculation results showed that OGD 10, 15, and 30 min caused significant percentage tissue injury in hippocampal slices compared with the control group (*F*_3,20_ = 8.867; 10 min: *P* = 0.0033; 15 min: *P* = 0.0013; 30 min: *P* = 0.0006), but there was no significant difference among these three OGD groups. The previous studies have shown that moderate and reversible damage in hippocampal tissue was observed after OGD for 15 min (Zhang et al., 2008; Hernández-Guillamon et al., 2014). Therefore, OGD for 15 min was chosen to use in the subsequent experiments. The concentration-response relationship in the effects of cordycepin on hippocampal tissue injury induced by OGD was investigated. We observed that cordycepin-pretreated at dosages of 20 μg/ml significantly decreased the damages of hippocampus caused by OGD (*F*_3,14_ = 3.217, *P* = 0.0473; Figures 6C,D). Therefore, a cordycepin of 20 μg/ml was chosen to use in the subsequent experiments.

**Figure 7:**
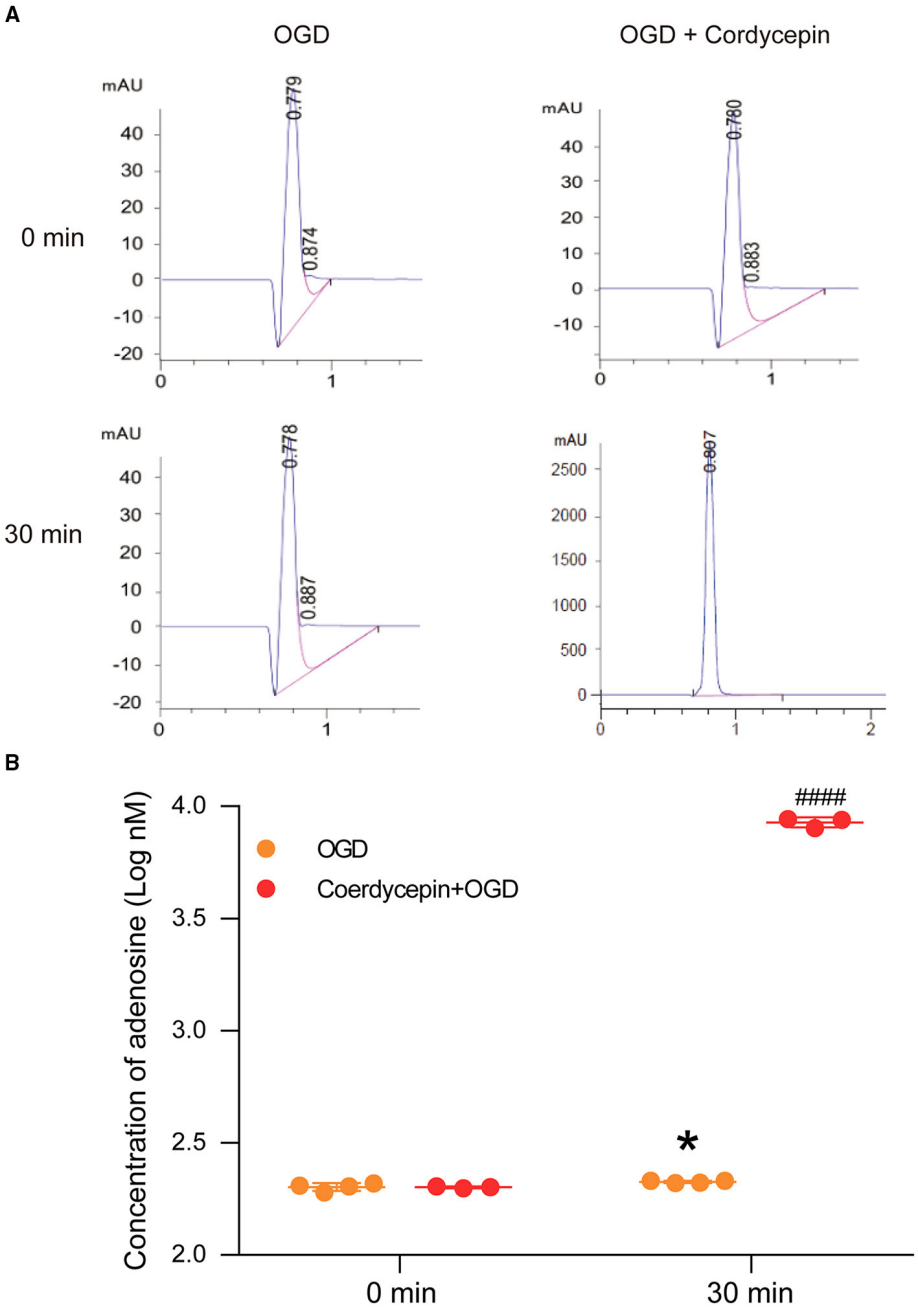
Cordycepin increased the level of adenosine in hippocampal slices after OGD. **(A)** Representative analysis diagrams of adenosine level from OGD and cordycepin + OGD groups for 0 min and 30 min. **(B)** The content of adenosine. OGD increased the content of adenosine, and pre-perfusion of cordycepin markedly increased the content of adenosine after OGD. OGD group *n* = 4 mice, cordycepin + OGD group *n* = 3 mice. Data were expressed as mean ± SEM; Student's *t*-test, *p < 0.05, OGD 0 min vs. OGD 30 min, ^####^*p* < 0.0001, OGD 30 min vs. cordycepin + OGD 30 min.

The perfusion fluids from OGD and cordycepin + OGD groups were collected at two-time points, including 0 min (baseline, before OGD) and 30 min (after treatment of OGD or cordycepin + OGD), to detect the content of adenosine. We observed that the concentration of adenosine was significantly increased after OGD, as shown in Figures 7A,B (*t*_6_ = 2.656, *P* = 0.0377). Interestingly, in the cordycepin + OGD group, the concentration of adenosine was also significantly improved compared with the OGD group (*t*_5_ = 39.89, *P* < 0.0001; Figure 7B). The results demonstrated that cordycepin further improved the content of adenosine after OGD.

### Blocking A1R Rather Than A2AR Diminished the Protection Role of Cordycepin on Synaptic Transmission Against OGD

**Figure 8:**
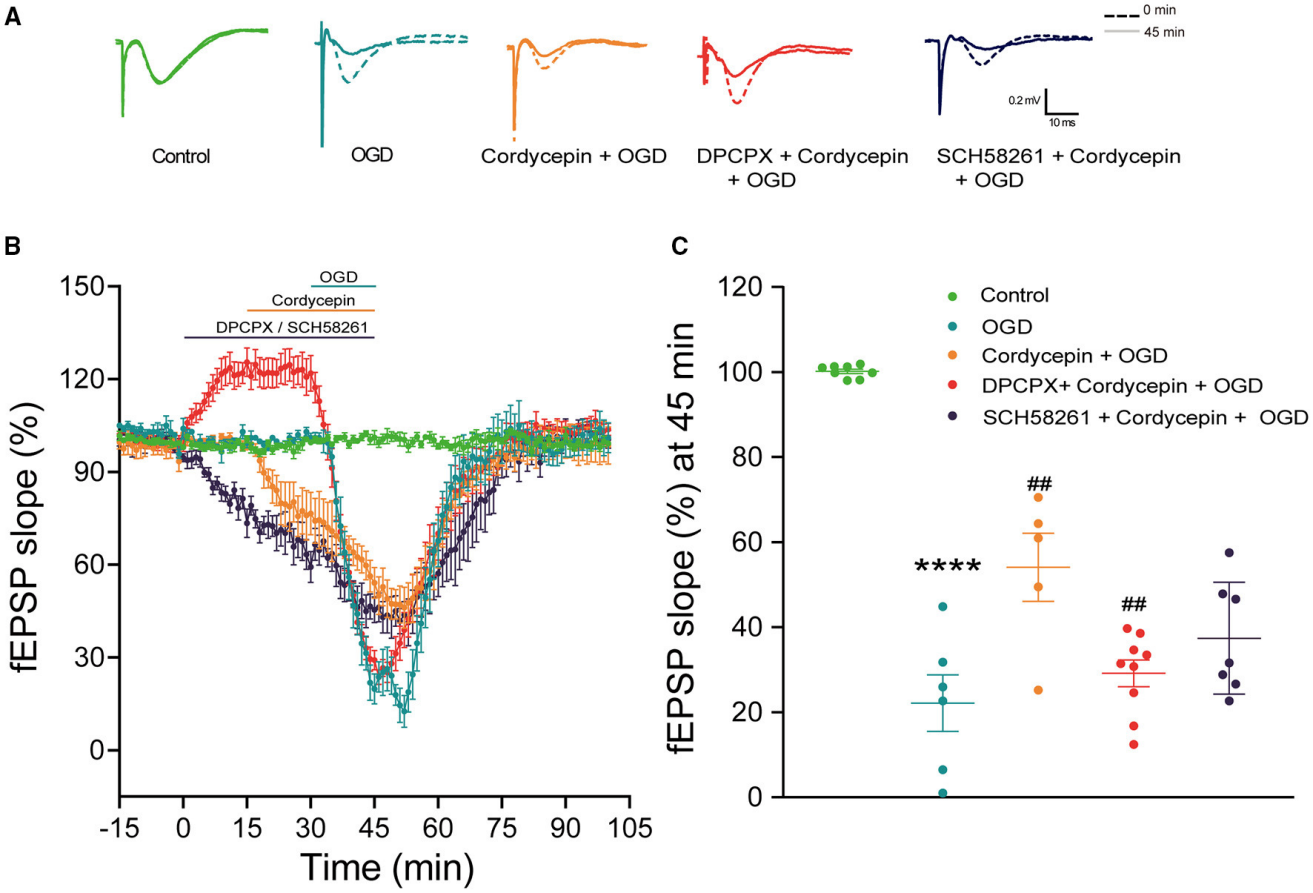
Blocking A1R rather than A2AR diminished the protective role of cordycepin on synaptic transmission against OGD. **(A)** Representative records of the evoked potentials from the control, OGD, cordycepin + OGD, DPCPX + cordycepin + OGD groups and SCH 58261 + cordycepin + OGD groups; The dotted line: 0 min; The solid line: 45 min. **(B)** The fEPSP slope was observed in the control, OGD, cordycepin + OGD, DPCPX + cordycepin + OGD groups and SCH 58261 + cordycepin + OGD groups. **(C)** Effects of DPCPX and SCH 58261 on the synaptic transmission after OGD and cordycepin pretreatment. The numbers of control, OGD, cordycepin + OGD, DPCPX + cordycepin + OGD and SCH 58261 + cordycepin + OGD groups were 8, 6, 5, 9, and 7 mice, respectively. Data were expressed as mean ± SEM; one-way ANOVA,****p < 0.0001, OGD vs. cordycepin + OGD; ^**##**^*p* < 0.01, cordycepin + OGD vs. DPCPX + cordycepin + OGD or SCH 58261 + cordycepin + OGD. DPCPX, the antagonist of A1R; SCH 58261, the antagonist of A2AR.

The previous research has proved that 20 μg/ml cordycepin significantly decreased fEPSP and recovered quickly after washout (Yao et al., 2013). Likewise, cordycepin alone decreased the slope of fEPSP in 15–30 min in the present study, representative records of the fEPSP slope were shown in Figure 8A. We further observed that the fEPSP slope was significantly reduced in hippocampal slices exposed to OGD, and then recovered gradually to the baseline level after about 45 min (*F*_2,16_ = 65, *P* < 0.0001; Figures 8B,C). However, the OGD-induced decrease in fEPSP slope in the 20 μg/ml cordycepin + OGD group was inhibited compared with the OGD group (*F*_2,16_ = 65, *P* = 0.0015; Figures 8B,C). These findings demonstrated that the pre-treatment of cordycepin could reverse the reduction of synaptic transmission induced by OGD.

In order to explore whether adenosine receptors were involved in the above process, hippocampus slices were exposed to the selective antagonists of A1R or A2AR in advance. As expected, DPCPX (the antagonist of A1R, 2 μM) (Li et al., 2011; Zhang et al., 2012) alone increased the fEPSP slope. In Figure 8B, the improved effect of cordycepin on the decrease of fEPSP amplitude induced by OGD was offset by DPCPX and decreased to the same level as the OGD group. Figure 8C showed that the slope of fEPSP at 45 min markedly reduced as compared to the cordycepin + OGD group (*F*_2,18_ = 5.953, *P* = 0.0053; Figure 8C). However, at the presence of SCH 58261 (the antagonist of A2AR, 500 nM) (Ferguson and Stone, 2010), the fEPSP slope had no difference compared with the cordycepin + OGD group (*F*_2,18_ = 5.953, *P* = 0.0709; Figures 8B,C). These results indicated that the protective role of cordycepin in synaptic transmission against OGD was almost eliminated by the antagonist of A1R.

## Discussion

The present study displayed that cordycepin reduced dendritic morphological damage and improved behavioral LTP in GCI rats, accompanied by the reversal of A1 receptors downregulation. Cordycepin also provided the improvement on synaptic transmission by regulating A1R in the OGD hippocampal slices. These findings provided new insights into the pharmacological mechanisms of cordycepin for ameliorating cognitive impairment induced by cerebral ischemia.

Global cerebral ischemia (GCI) has been widely studied using two-vessel occlusion (2VO), three-vessel occlusion (3VO), and four-vessel occlusion (4VO) rodent models (Liu et al., 2012; Pereira et al., 2012). However, 3VO and 4VO models led to several risks, including pneumothorax occurs, spinal cord damage, and post-ischemic seizures (McBean and Kelly, 1998). 2VO model is closer to an attack of transient forebrain hypoxic-ischemic disease in humans (Hartman et al., 2005) and has been widely used because of its less trauma and simple operation. To avoid the occurrence of hind limb dysfunction caused by blood withdrawal (Kristian and Hu, 2013), antihypertensive drugs, such as phentolamine and sodium nitroprusside, were used to compensate for incomplete ischemia in the 2VO model in our experiments. We found that 2.5 mg/kg sodium nitroprusside could guarantee the appropriate hypotension and duration of maintenance. Additionally, our results showed that 2.5 mg/kg sodium nitroprusside combined with two vessel occlusions for 20 min caused about 50% CA1 neuronal death and learning and memory impairments in the Y maze test. Similarly, many studies showed that more than 50% loss of hippocampal CA1 neurons was more likely to lead to cognitive impairment (Hartman et al., 2005; Yang et al., 2017).

Synaptic transmission and synaptic plasticity are the essential processes of brain physiological functions for learning and memory. Synaptic transmission is the communication between presynaptic and postsynaptic neurons and the subsequent processing of the signal (Wei et al., 2015). Synaptic changes occur early after cerebral ischemia, reflecting the pathologic changes associated with synaptic transmission (Neumann et al., 2013). OGD is a widely used model to mimic cerebral ischemia *in vitro*. OGD for 15 min was selected in our experiments because a previous study reported that the damage of hippocampal pyramidal cells was functionally recoverable after OGD for 15 min (Zhang et al., 2008). Our results showed that OGD for 15 min markedly reduced the fEPSP slope, and then recovered gradually to the baseline level after about 45 min (Figure 8B), which is consistent with previous findings that 15 min OGD protocol induced a continuous and significant depression of synaptic transmission at CA3 MF and AC synapses but not at CA1 SC synapses and an alternative possibility is local differences in the coupling of A3R or mGluR1 to their associated G-protein receptors (Dixon et al., 2009; Dennis et al., 2011). In addition, the synaptic depression caused by OGD 15 min was significantly restored by hypothermia (32 or 28°C) (Oyama et al., 2020), which was also observed at 32°C in our experiment.

Furthermore, studies have shown that LTP damage induced by cerebral ischemia in the hippocampus is associated with learning and memory impairments (Hammond et al., 1994; Takeuchi et al., 2015). LTP is an important manifestation of long-term synaptic plasticity and is considered the foundation of learning and memory (Prieto et al., 2017). There are many ways to induce LTP in the hippocampus, the most common of which is high-frequency stimulation (HFS). However, the LTP electrophysiological record of this method is separate from the behavioral test (Min et al., 2017). Behavioral LTP, an LTP model induced by behavioral training, is commonly used to study long-term synaptic plasticity induced by learning and memory tasks in sober animals. At the technical level, the bigger real problem is that behavioral-LTP technology has not yet made a breakthrough in mice, and comparatively speaking, embedding electrodes in the brain area of rats and administering drugs can bring more operating space to the behavioral-LTP technology. Thus, we adopted the rat model to further study the relationships between cordycepin, behavioral LTP, and adenosine A1 receptors. In addition, the distribution of adenosine A1 receptors in the brains of rats and mice is similar (Fastbom et al., 1987), the affinities of its antagonist (DPCPX) are also similar (Alnouri et al., 2015).

Our study showed that the behavioral LTP magnitude and the learning and memory abilities of GCI rats were impaired simultaneously, and cordycepin could reverse learning and memory impairments and behavioral LTP damage. These results suggested that the effects of cordycepin on synaptic transmission and synaptic plasticity could contribute to its improvement on learning and memory in cerebral ischemia.

The structural plasticity of dendrites and spines is widely related to synaptic function and learning and memory (Sala and Segal, 2014; Bailey et al., 2015). They receive neural signals and form synapses, which are key parts of neuronal communication. The hippocampal CA1 region is highly vulnerable after cerebral ischemia and is more sensitive than other hippocampal areas (Lu et al., 2016; Li et al., 2017). Our results showed that GCI caused dendritic atrophy and reduced dendritic spines in the hippocampal CA1 region. Previous studies have revealed that irreversible dendritic damage and spines loss occurred within 10–20 min ischemia and hypoxia (Brown and Murphy, 2008), resulting in a significant reduction in dendritic branches and dendritic spines (Rojas et al., 2013; Zhu et al., 2017), which are consistent with our findings. Adenosine, an important neuromodulator in the nervous system, could regulate the function of the synaptic transmission by activating adenosine receptors (Fredholm et al., 2005). Four adenosine receptors are containing A1, A2A, A2B, and A3 receptors. A1R and A2AR are shown a widespread distribution in the brain and are closely associated with cerebral ischemia (Ribeiro et al., 2002). Limited literature showed that A2B and A3 receptors play a role in regulating inflammation and excitotoxicity in cerebral ischemia due to their low level in the brain (Ham and Rees, 2008; Pedata et al., 2016). Cordycepin may inhibit the production of pro-inflammatory cytokines through the A3 receptor to produce anti-inflammatory effects (Du et al., 2021) to improve cerebral ischemia damages, but it is unlikely to speculate from our present results that cordycepin aggravated the synaptic failure caused by OGD through A3 receptors (Pugliese et al., 2006) or A2B receptors (Fusco et al., 2018). Furthermore, activating A1R protected neurons against neurotoxicity and attenuated the memory impairments induced by ischemia-reperfusion (Ribeiro et al., 2002; Zamani et al., 2013). In the present study, cordycepin increased adenosine A1R level but showed no effect on the A2AR level, which resembled our previous findings (Dong et al., 2019). There are convincing indications that adenosine receptors control dendritic morphology and markers. Since the activation of A2AR was previously reported to increase dendritic branching (Ribeiro et al., 2016). Caffeine, a non-selective antagonist of A1R and A2AR, prevents the loss of nerve terminals (Alves et al., 2020) and increases the number of neurons with more branch points, total and maximal neurite length (Duarte et al., 2012). Interestingly, the A2AR agonist (CGS 21680) promoted more neurite branching *via* PKA signaling, A2AR antagonist (SCH 58261) contributed to axonal outgrowth through PI3K but not PKA signaling (Alves et al., 2020). In the present study, cordycepin treatment alleviated the damage of dendritic branches and spines in the hippocampal CA1 area caused by cerebral ischemia, which may be related to the results of regulation of A1R by cordycepin, and the specific mechanism and related pathways need to be further studied. Although the inhibition constant (Kd) value of DPCPX (the antagonist of A1R) for adenosine Al receptors in the hippocampus is 0.45 nM (Sebastião et al., 1990), a high concentration (2 μM) of DPCPX (the antagonist of A1R) was applied to block increased adenosine Al receptors induced by cordycepin. Moreover, DPCPX, a selective antagonists of A1R facilitated synaptic transmission, increased the fEPSP slope as reported previously (Bon and Garthwaite, 2002; Brust et al., 2006), dismissing depression of cordycepin on synaptic transmission in the first 30 min. Likewise, DPCPX (the antagonist of A1R) attenuated the protective effect of cordycepin on synaptic transmission in OGD, suggesting that cordycepin altered synaptic transmission *via* A1R. SCH 58261 (the antagonist of A2AR) significantly decreased fEPSP, which might be related to the decrease of presynaptic glutamate release by A2AR antagonists (Blum et al., 2003; Quiroz et al., 2009). In addition, at the presence of SCH 58261 (the antagonist of A2AR), the fEPSP slope in the SCH 58261 + cordycepin + OGD group has no difference compared with the cordycepin + OGD group at 45 min, which indicated that the protective role of cordycepin in synaptic transmission against OGD was not related to A2AR. Previous studies have suggested that extracellular adenosine concentration is increased significantly after cerebral ischemia, and adenosine protected against excitotoxicity by activating A1R (Saransaari and Oja, 2002; Pearson et al., 2006). These supported our results that the level of adenosine elevated after OGD and further markedly increased with cordycepin application. One of the reasons for the non-parallel changes in adenosine and adenosine A1R after cordycepin treatment may be related to the desensitization of A1R because A1R was prone to rapid desensitization in the presence of a high dose of adenosine (Von Lubitz et al., 1995). In addition, there was no significant change in the level of A2AR in the cordycepin group after GCI, which seemed to contradict the previous report that the level of A2AR increases accompanied with the supplement of exogenous adenosine in the hippocampal CA1 area after temporary cerebral ischemia (Seydyousefi et al., 2019). We speculated that the rapid increase of adenosine in the short term was maybe not enough to change the level of A2AR. A2AR is present at low levels in the hippocampus (Dixon et al., 1996), and its roles are quite complex in cerebral ischemia. For example, both activation or blockade of A2AR could enhance neuronal survived in cerebral ischemia (Pugliese et al., 2009; Maraula et al., 2013).

## Conclusion

The present study showed that cordycepin could alleviate learning and memory impairments after cerebral ischemia by protecting dendritic morphology and synaptic function, an action mediated by adenosine A1R. These findings would be helpful to share light on the mechanisms underlying the roles of cordycepin in ameliorating cognitive impairments induced by cerebral ischemia.

## Data Availability Statement

The raw data supporting the conclusions of this article will be made available by the authors, without undue reservation.

## Ethics Statement

The animal study was reviewed and approved by the Ethics Committee of Animal Research of South China Normal University.

## Author Contributions

C-HL supervised the study and designed the experiments. Z-HC and Y-YH performed the experiments. Y-JS and S-YZ analyzed the data. Z-HC wrote the manuscript. Y-YH revised the manuscript. J-NH and B-YW provided suggestions and assistance in the experiment design. All authors contributed to the article and approved the submitted version.

## Funding

This work was supported by the Guangdong Basic and Applied Basic Research Foundation (2019A1515012025) and the Scientific and Technological Planning Project of Guangzhou City (201805010002).

## Conflict of Interest

The authors declare that the research was conducted in the absence of any commercial or financial relationships that could be construed as a potential conflict of interest.

## Publisher's Note

All claims expressed in this article are solely those of the authors and do not necessarily represent those of their affiliated organizations, or those of the publisher, the editors and the reviewers. Any product that may be evaluated in this article, or claim that may be made by its manufacturer, is not guaranteed or endorsed by the publisher.
